# Crackling noise microscopy

**DOI:** 10.1038/s41467-023-40665-4

**Published:** 2023-08-16

**Authors:** Cam-Phu Thi Nguyen, Peggy Schoenherr, Ekhard K. H. Salje, Jan Seidel

**Affiliations:** 1 School of Materials Science and Engineering, UNSW Sydney, Sydney, NSW 2052 Australia; 2 ARC Centre of Excellence in Future Low-Energy Electronics Technologies (FLEET), UNSW Sydney, Sydney, NSW 2052 Australia; 3 Department of Earth Sciences, Cambridge University, Cambridge, UK

**Keywords:** Characterization and analytical techniques, Characterization and analytical techniques

## Abstract

Crackling noise is a scale-invariant phenomenon found in various driven nonlinear dynamical material systems as a response to external stimuli such as force or external fields. Jerky material movements in the form of avalanches can span many orders of magnitude in size and follow universal scaling rules described by power laws. The concept was originally studied as Barkhausen noise in magnetic materials and now is used in diverse fields from earthquake research and building materials monitoring to fundamental research involving phase transitions and neural networks. Here, we demonstrate a method for nanoscale crackling noise measurements based on AFM nanoindentation, where the AFM probe can be used to study the crackling of individual nanoscale features, a technique we call crackling noise microscopy. The method is successfully applied to investigate the crackling of individual topological defects, i.e. ferroelectric domain walls. We show that critical exponents for avalanches are altered at these nanoscale features, leading to a suppression of mixed-criticality, which is otherwise present in domains. The presented concept opens the possibility of investigating the crackling of individual nanoscale features in a wide range of material systems.

## Abstract (web summary)

The authors present crackling noise microscopy, a method for measurement of the crackling of individual nanoscale features based on AFM nanoindentation. They use it to investigate crackling noise and avalanches in the domains and domain walls of ferroelectric materials.

## Introduction

Crackling noise is a general physical phenomenon in material systems exposed to external stimuli (force, fields, etc.)^1–4^. These stimuli induce atomic-sized kink movements in the material’s crystal structure in the form of jerks or avalanches accompanied by crackling noise (actual sound waves). Similar concepts apply to many other systems, from crumpling paper^5^, various phase transitions in solid materials^6^, deformed crystals and porous materials^7^, and neural networks^8^, to the seismic activity found in earthquakes^9–11^. Renormalization group theory predicts that these diverse crackling systems approach fixed points, indicating universal scaling behaviour^4^. For example, various types of universality classes can be found in crackling noise statistics, associated power laws, and scaling functions and can be studied with various macroscopic techniques^2^. Identification of universality classes enables efficient prediction of material properties in a wide range of applications^12^.

Indentation is one way to produce crackling noise in materials^13,14^, in addition to compression and bending^15,16^. Beirau and Salje investigated crackling noise avalanches in indentation measurements of mesoporous synthetic silica^14^, showing that mechanical properties are a function of depth and sensitive to chain reactions of collapsing pores during the indentation process. The crackling noise was shown to be power-law distributed with exponents of approximately 1.5 over several decades, confirming avalanche criticality. One drawback of conventional indentation is that individual nanoscale features cannot be chosen for investigation, as these systems do not incorporate imaging of materials surfaces. A solution to this problem is to use a scanning probe system capable of various imaging modes to locate and study nanoscale features prior to indentation. With the availability of calibrated hard solid diamond probes and rigid, stable scanners for long indentation time to record sufficient statistical data of >10^3^ individual jerk events occurring typically within 10^−4^ s^4^, this concept appears feasible.

Here, we examine the ability to use AFM-based nanoindentation to investigate crackling noise in ferroelectric lead titanate (PbTiO_3_) as a test material system. A maximum loading force of 30 µN is applied to the surface over a long time (hours) through a single crystal diamond AFM cantilever. During that time, we detect the signal produced by any surface movements. Individual movements are typically in the sub-Å to pm range and at the limit of the AFM’s sensitivity. To determine the twin domains and domain walls as well as the difference in mechanical response of different area, we used other AFM-based imaging techniques which are well-defined in our current reports^17,18^. While crackling noise has been investigated in the context of ferroelectric materials^19–21^ in macroscopic and bulk measurements, our technique provides insight into the single topological defect level. The test material PbTiO_3_ can be considered a prototypical tetragonal ferroelectric system^22–24^, that has been shown to possess functional topological defects, i.e. conductive domain walls with intrinsically different nanoscale material properties from the bulk material^18,22,25^. Due to the structure of the tetragonal phase, both 90° and 180° domains exist in ferroelectric PbTiO_3_ single crystals^18,24,26–28^. The difference between the in-plane (IP) and out-of-plane (OOP) domains is a rotation of the c-axis by 90°. This leads to the twinning of the material and twin boundaries separating the IP and OOP domains. In this research, the crackling noise in a variety of regions on a PbTiO_3_ single-crystal sample has been investigated, including twin domains (i.e. IP and OOP domains) and 180° domain walls.

## Results

### The measurement concept

**Fig. 1:**
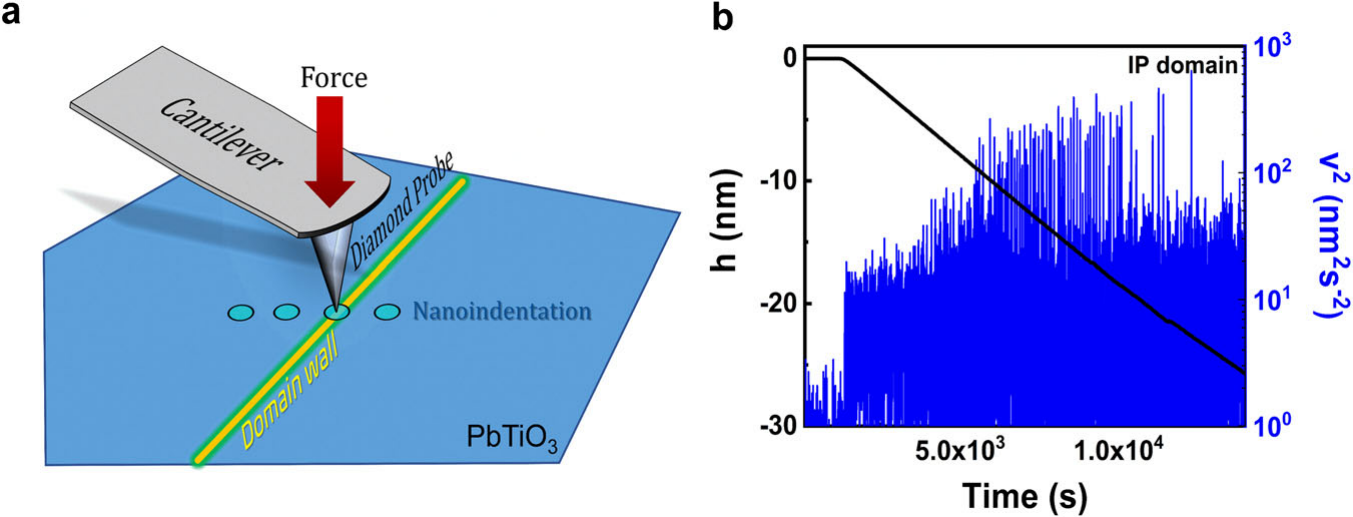
Crackling noise detection based on AFM nanoindentation. **a** A constant force, typically in the nN range and depending on material hardness, is applied over a long period (hours) through an AFM probe and surface movement is detected at the limit of the AFM’s sensitivity, typically in the sub-Å to pm range, depending on the specific setup. Individual nanoscale features, such as domain walls in ferroelectrics, can be selected prior by other AFM-based imaging techniques which are well-defined in our current reports^17, 18^. **b** Example of a recorded avalanche distribution under the AFM probe.

A jerk is defined as an individual spike in the crackling noise spectrum. Jerk energy can be represented by the squared time derivative of the height (d*h*/d*t*)^2^ measured through an AFM probe (Fig. 1a). In AFM-based indentation, the AFM probe in contact with the sample is simultaneously used to apply a force and to measure the reaction of the material under the tip in the form of an indentation depth. Figure 1b presents an example of results detected by the AFM probe during one indentation measurement. The black line displays the measured indentation depth *h(t)*, and the blue line shows the squared velocity *v*^*2*^*(t)* = (d*h*/d*t*)^2^. Both curves are shown versus time while increasing the force exerted by the AFM probe from 0 to 30 µN with a very low rate of 2.08 nN/s. At low loading force, the AFM probe does not create indents on the sample surface. Thus, the indentation depth is zero. When the force is strong enough, the AFM probe generates surface deformation and jerks. The indentation depth and jerk intensity are increased with the increase of force. It can be seen as a shift of the height value to the negative side, and the jerky evolution of the indentation depth is apparent. This data is the key spectrum for all of the following avalanche analyses.

The probability distribution of the jerk energy can be extracted from an indentation process by analyzing the indentation depth of a loading curve. A reliable statistical analysis of the crackling noise is generated by a large data set, typically with at least ~10^3^ jerks induced by an increasing force on the cantilever. On a log–log plot, the energy distribution *g*(*E*) of jerks is a function of the energy *E* of avalanche events and is given by refs. ^21,29,30^:

1 $$g(E)dE=\bigg(\varepsilon -1\bigg){\left(\frac{E}{{E}_{\min }}\right)}^{-\varepsilon }\frac{dE}{{E}_{\min }}\,E \, > \, {E}_{\min }$$

where *E*_min_ is the lower cutoff needed for normalization, and *ε* is the energy exponent.

The energy exponent describes the universal power laws of the crackling noise and can directly be extracted from the jerk energy distribution. A power-law dependence indicates that the crackling behaviour only depends on symmetries or dimensions of the material and is independent of microscopic details^11^. However, measured crackling noise can have several sources or be distorted by exponential damping factors that do not allow for the direct extraction of the exponent from the distribution of the jerk energy. The maximum-likelihood *ε* (ML) fitting method that estimates the exponent ε above a fixed jerk energy value *E*_0_ can be used to identify deviations from a unique crackling noise source^21,29,30^. The exponent *ε* is given by:

2 $$\varepsilon ({E}_{\min })=1+n{\left(\mathop{\sum }\limits_{i=1}^{n}{{{{\mathrm{ln}}}}}\frac{{E}_{i}}{{E}_{\min }}\right)}^{-1}$$

It is important to note that not all jerks are manifestations of avalanches, they can also be produced by the measuring device or sample vibrations. Therefore, such artifacts have to be eliminated before avalanche statistics based on a jerk spectrum can be discussed. Soprunyuk et al.^11^ reported that jerks with squared velocities less than 10 nm^2^ s^−2^ do not contribute to the power-law behaviour and behave like white noise. Moreover, strain rates of 10^−5^ /s are typically required for slip avalanche experiments on crystal materials, as discussed by Salje et al.^2^. This indicates that observation time plays an important role in the avalanche experiment. The time should be long enough to obtain high-quality jerk measurements with reliable statistics^2^.

**Fig. 2:**
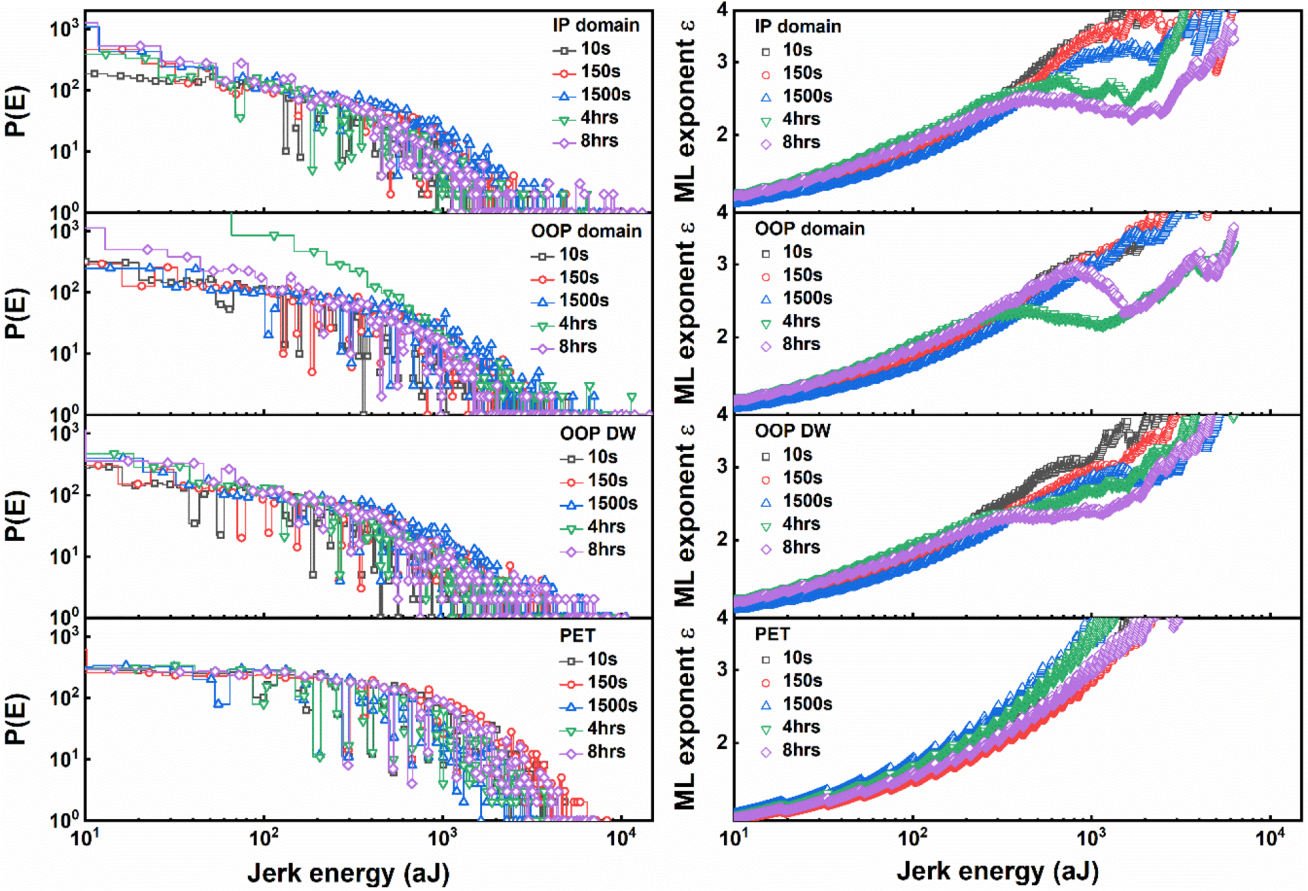
Crackling noise detection based on AFM nanoindentation.Time-dependent measurements of the probability distribution of jerk energy *P(E)* and the maximum-likelihood critical exponent *ε*. *P(E)* and *ε* of in-plane (IP) domains, out-of-plane (OOP) domains, OOP domain walls (DWs) of PbTiO_3_ single-crystal sample, and polyethylene terephthalate (PET) sample.

In our experiment, we thus first investigate the crackling noise as a function of different indentation periods of 10 s, 150 s, 1500 s, 14,400 s (4 h), and 28,800 s (8 h), i.e. the time used to measure one indentation cycle. The total number of data points in each cycle (including loading and unloading curves) is independent of the indentation period and was set to 10^4^. Figure 2 shows time-dependent measurements of probability distribution of jerk energy *P(E)* and the ML exponent ε acquired on the IP domain of a single crystal PbTiO_3_ sample. The *P(E)* curves look roughly similar independent of indentation speed or sample location, which makes it difficult to determine the energy exponent. The ML curves accentuate the exponent *ε* and it can be seen that the PbTiO_3_ shows a clear difference from the PET samples. This is a clear indication that there is crackling noise occurring in the material. At indentation times less than 1500 s, the ML curves are the same as the polymer ones and do not show any plateau or deflection. Meanwhile, for indentation times between 1500 s and 8 h, there is a clear deviation from the curves indicating the existence of a jerk energy distribution of avalanches in the material. These results suggest that the jerks are only observable when the indentation time is long enough to distinguish clearly between individual jerks^2^.At indentation times less than 1500 s, a slight change of ε around 10^3^ aJ is visible. For longer indentation times between 1500 s to 8 h, there is a clear deviation and cut-off indicating the existence of a jerk energy distribution of avalanches in the material. This is the first indication that the crackling noise occurring in the material can be successfully recorded with our AFM-based method. These results suggest that the jerks are only observable when the indentation time is long enough to distinguish clearly between individual jerks^2^. Therefore, for further investigation, we used the longest indentation time of 8 h to analyse and compare the crackling noise of different PbTiO_3_ regions.

In consideration of the above, the validity of our measurement concept was tested by comparative measurements on viscoelastic materials that creep, i.e. deform continually as long as the stress is present, which is the case for many polymer materials^31^. Detailed data of measurements on polyethylene terephthalate (PET, see Fig. 2), polypropylene (PP), and polystyrene (PS) can be found in Fig. S1 of the supplementary material. The utilization of high viscoelastic polymer materials allows us to test for artifacts in the measurements (laboratory and system noise, sample vibrations). As shown in Fig. 2b and the Supplementary material, all polymer samples reveal a similar behaviour independent of their indentation times. It is harder to see the difference from the probability distribution of jerk energy *P(E)* plots, but the ML curves of the PbTiO_3_ exhibit a clear exponent *ε* with a kink and plateau which does not appear in the polymer samples. No cut-off energies and avalanches occur in the polymer samples, thus showing that our AFM-based method is working properly without artifacts impacting the measurements.

**Fig. 3:**
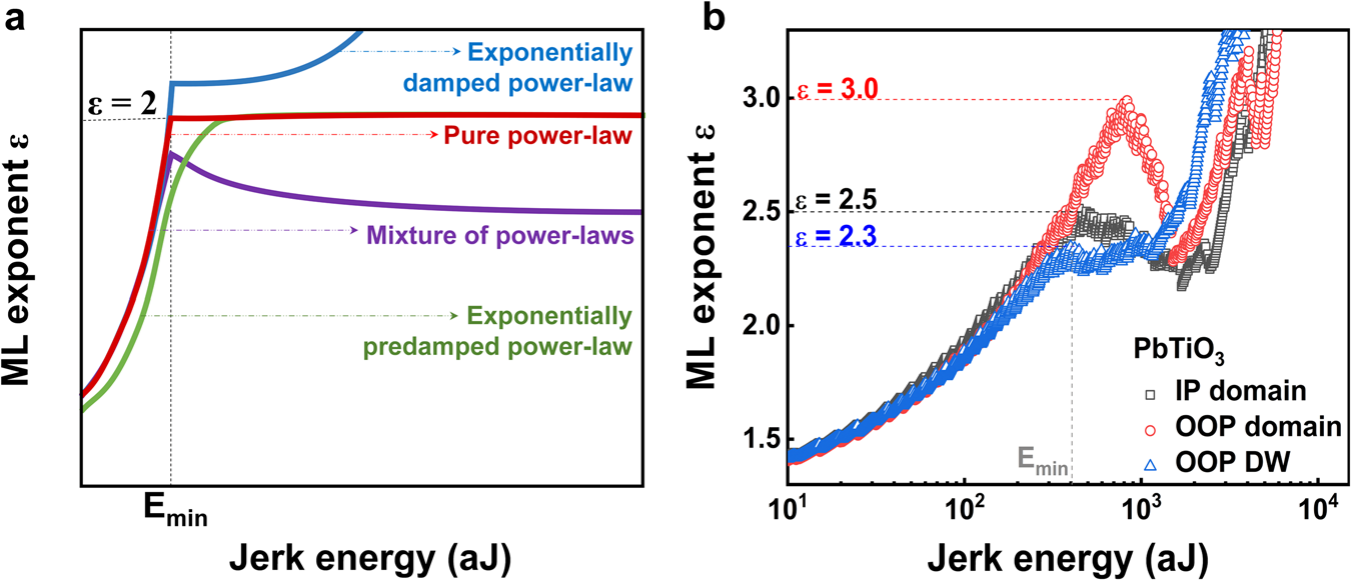
Mixed and damped critical power-law contributions at PbTiO_3_ domain walls. **a** Different ML fitted exponent *ε* behaviour. **b** ML fitted exponent *ε* for crackling noise measurements at OOP, IP domain, and 180° OOP domain wall in PbTiO_3_. Dashed lines indicate averaged exponents at medium jerk energies. All ML curves coincide at 2.3 near *E* = 2 × 10^3^ aJ which represents the lower common critical exponent.

In the case of a single exponential crackling noise source, the ML exponent *ε* stabilizes at a fixed value for higher jerk energies (*E* > *E*_min_). This perfect behaviour is represented by the red line in the graph of Fig. 3a that is calculated for synthetic generated crackling noise values with exponent 2. The calculation has been reported by E.K.H. Salje et al.^9^. Below *E*_min,_ there is a kink and a fast decrease of the exponent *ε* that is justified by the reduced number of low jerk energies that are found in experiments coming from the limitations of the measuring system. The other three curves show examples of mixing of two exponents (purple line), predamping (green line) and exponential damping (blue dotted line). This understanding of the ML curves can be used to analyse our experiments on PbTiO_3_.

### Crackling noise of domains and domain walls

Figure 3b shows the measured ML curves of the IP and OOP twin domains and 180° OOP domain wall of the PbTiO_3_ sample with an indentation time of 8 h. Both domains and domain wall show a clear discontinuity in the ML slope which is the signature of avalanches in PbTiO_3_. All the ML curves show an increase in exponent *ε* with increasing energy until *E*_min_, where a sharp kink appears. This cut-off energy of 4.09 × 10^2^ aJ is the same for IP domain and OOP domain wall while the OOP domain has a higher apparent cut-off energy at about 8.15 × 10^2^ aJ but there is a small break of slope at lower energies at about 4.09 × 10^2^ aJ as same as the IP domain and OOP domain wall. This result is reasonable as they were measured with the same cantilever and system. For higher jerk energies the ML curves show a certain level of plateauing with a typical increase of *ε* at higher jerk energies. Interestingly, the shape of the plateau is different for the IP domain, OOP domain, and 180^o^ OOP domain wall. For the 180° OOP domain wall, there is a plateau at a value of 2.3, whereas ε of the OOP domain reaches up to 3.0 and then falls back to about 2.3, where ε for the IP domain only goes up to 2.5 and then falls back to 2.3. This behaviour of both domains is typical for a mixture of two power-law contributions as reported by Salje et al.^9^. If a mixture of power-law contributions exists, the ML curve normally does not show a pure plateau but varies between two power-law contributions. This can also depend on how strong the mechanism is behind each of them. Therefore, analyzing the ML curves on different PbTiO_3_ areas shows that there is a common exponent of 2.3 in each of the measurements with an additional contribution for the OOP and IP domains. Model simulations^9^ have shown that the lower exponent is readily seen in the experimental ML curves while the upper exponent is much harder to identify. The maximum of the ML curves represents a lower bound of the upper exponent. Our results confirm this mechanism by showing a common exponent of 2.3 for all samples which can be taken as the characteristic bulk value for mesoscopic twin movements^32–34^. The larger additional exponent in the domains could relate to different avalanche movements and may include surface effects.

Typically, the energy exponent *ε* varies between 1 and 3^2,4,13,30,35^ for crackling noise investigated by macroscopic and bulk measurements. In our measurement, we enable using AFM-based nanoindentation as a driver for crackling noise to analyse microscopic areas on a ferroelectric material. The results reveal higher crackling noise values for the IP and OOP domains than expected for the mean-field solution (1.33 and 1.66)^2^. Enhanced exponents were also found in complex twin structures reported by Casals et al.^29^. These jerks occurring in twin reconfigurations induced by an electric field follow a power law with a strongly increased damping and hence, have a larger energy exponent (*ε* = 1.6) compared to the simple twin pattern (*ε* = 1.4). Additionally, the twinning in metals^36,37^, and the jerky movement of ferroelastic domain walls of PbZrO_3_ and LaAlO_3_^38^ show exponents near 1.8–2.0. Equally, the mixture of power laws with high exponents was observed in martensitic transitions^39^ where the upper exponent was also reported as *ε* = 2. Therefore, the higher exponent values *ε* in the IP and OOP domains are reasonable due to the complex structure of the twin domains.

Moreover, from a more general perspective, avalanches and criticality have also been studied in other topological defects than domain walls, including skyrmions^40^ and dislocation-heavy metals^41^. Díaz et al.^40^ reported avalanches with power-law distribution of both size and duration, with exponents of 1.55 and 1.63. For a larger value of the considered Magnus term, the average avalanche shape became strongly asymmetric and indicated that the observed effective negative mass was similar to avalanche distribution in domain walls. Additionally, dislocations as another form of topological defect have also been investigated^41^ showing the coexistence of two deformation modes of conventional thermally-activated smooth plastic flow and sporadic bursts. Dislocation avalanches in bcc metals have revealed an extended dislocation-avalanche velocity-relaxation.

In this study, we demonstrate a method for the crackling of individual nanoscale features based on AFM probe, a technique we call crackling noise microscopy. The method is successfully applied to investigate crackling noise and avalanches in the ferroelectric domains and domain walls of ferroelectric PbTiO_3_. The indentation time plays an important role as it needs to be long enough to distinguish every single jerk of the avalanches. Analyzing the energy distribution of the jerks and maximum-likelihood exponent curves allow for the identification of unique crackling noise at domains and domain walls of PbTiO_3_ single crystal. The energy exponents of the different PbTiO_3_ areas show a common value of 2.3 in each of the measurements with an additional contribution for the twin domains.

The presented concept opens the possibility of investigations of the crackling of individual nanoscale features in a wide range of material systems. Samples with ferroic patterns, such as complex twinning, could be analyzed on a local scale of some estimated 100 × 100 × 50 nm. In this region, the presence of a single twin boundary with a width of approximately 2 nm leads to a 2% increase in the internal jerk spectrum. When multiple twin boundaries act collectively, the resulting signal is much stronger than that of a single boundary, resulting in significantly larger signals in a dense pattern. The dense twin patterns show twin walls with distances less than 20 nm and the jerk contribution, therefore, raises to more than 10% which is likely to be seen by the mixing procedure. In this case, the response of the twin walls to the AFM stress is a sideways movement, its contribution increases steeply and our method should be ideal for such investigations.

## Methods

PbTiO_3_ single crystals were grown from PbO and TiO_2_ precursors (Sigma-Aldrich, high-purity 5 N (99.999%). The precursors have been first ground to powder in an agate mortar. Powder PbO and TiO_2_ are then synthesized in a sealed quartz crucible (10^−6^ mbar) by the solid-state reaction method for 48 h at 850° C in a horizontal muffle furnace. The purity of the synthesis of PbTiO_3_ powder was verified by X-ray diffraction^42,43^ and then put into a platinum crucible with additional PbO in molar ratios of 1:1 to 1:3. The crucible was covered to avoid PbO evaporation during crystal growth. The whole system is then placed in a cubic muffle furnace and heated up to 1200 °C in a period of 5 h. After 10 h at 1200 °C, the system is cooled down to 550 °C for a duration of 60 h. Further cooling to room temperature was performed at a cooling rate of 1 °C/h to avoid cracking at the phase transition from cubic to tetragonal (490 °C)^44–46^. Finally, hot acetic acid was used to extract the PbTiO_3_ from the PbO molten mass.

The AFM-based nanoindentation was performed by a Smart SPM 1000 system (AIST-NT, USA) using an NM-RC-C diamond probe with a force constant *k* = 470 N/m (Bruker, USA). The precise spring constant of each cantilever was determined using the thermal-noise technique^47,48^. The cantilever was calibrated on a fused-silica standard sample to determine its accuracy.

### Supplementary information

Supplementary Information

## Data Availability

Source Data file has been deposited in Figshare under accession code DOI link^49^.
