# Supplementary material for: Crackling noise microscopy
Source: Nat Commun. 2023 Aug 16;14:4963. doi: 10.1038/s41467-023-40665-4 (PMC10432464; doi:10.1038/s41467-023-40665-4)
Supplement: Supplementary file 1 — Supplementary Information [file 41467_2023_40665_MOESM1_ESM.pdf]

Supplementary Material

Crackling noise microscopy

Cam-Phu Thi Nguyen[1†], Peggy Schoenherr[1,2†], Ekhard K.H. Salje[3*], and Jan Seidel[1,2*]

[1]School of Materials Science and Engineering, UNSW Sydney, Sydney NSW 2052, Australia

[2]ARC Centre of Excellence in Future Low-Energy Electronics Technologies (FLEET), UNSW Sydney, Sydney 2052, Australia

[3]Department of Earth Sciences, Cambridge University, Cambridge, UK

[†]These authors contributed equally to this work

*Corresponding authors. E-mail: es10002@cam.ac.uk (E.S.); jan.seidel@unsw.edu.au (J.S.)

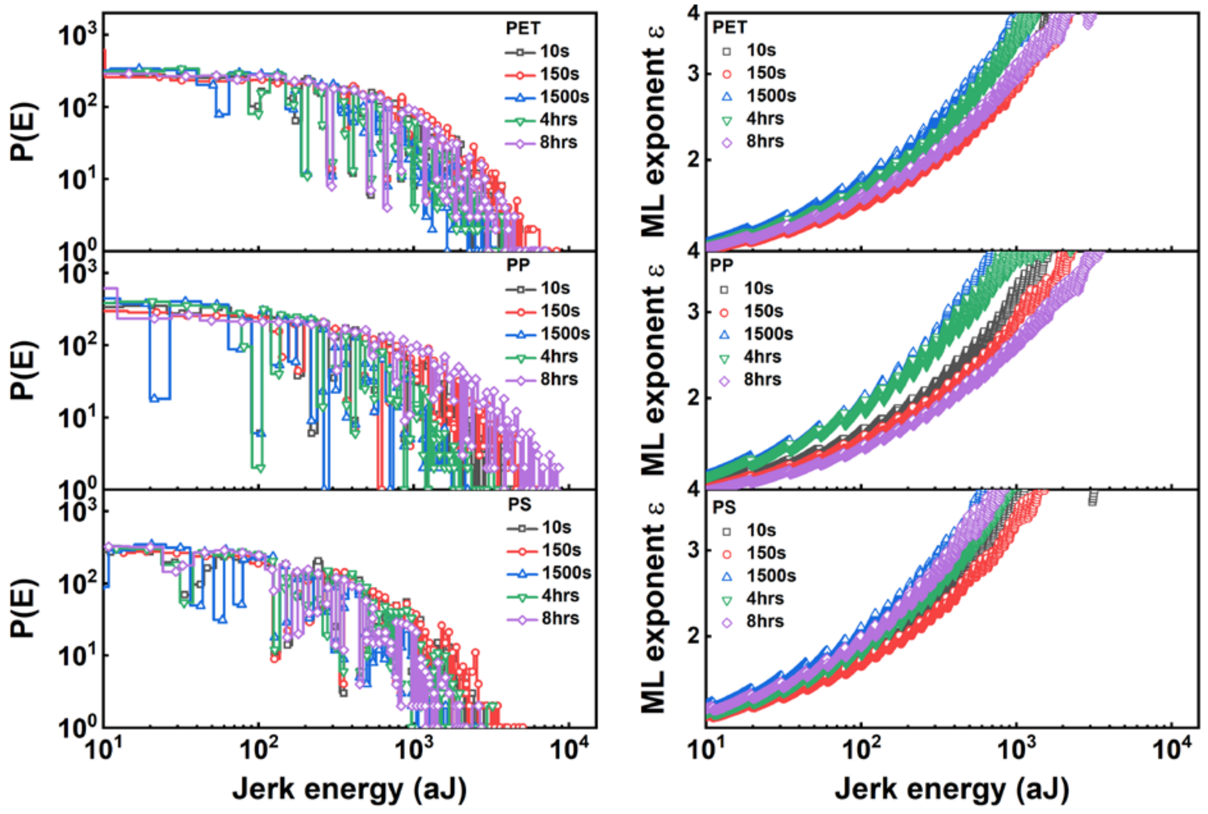

Fig. S1. Histogram of the probability distribution of jerk energy *P(E)* and maximum likelihood (ML) exponent curves of various types of polymers, including polyethylene terephthalate (PET), polypropylene (PP), and polystyrene (PS).

Polymers are viscoelastic materials that creep, i.e. deform continually as long as the stress is present [1]. Thus, those materials should not exhibit any crackling noise. This will allow us to extract any problems or artifacts with the measurements (laboratory and system noise, sample vibrations). The jerk energy distribution *P(E)* and maximum likelihood (ML) exponent ε of (PET, PP, PS) are presented in Fig. S1. As shown in Fig. S1, the polymer samples all reveal a similar behavior independent of indentation times. Harder to see from the *P(E)* representation, but clearly to make out in the ML graph, there is no clear exponent ε showing up in the measurements. A kink and plateau would appear in the ML curve if there was a defined crackling noise appearing in the material. This means no cut-off energies and avalanches occur in the polymer samples and our AFM-based methods are working with no additional artifacts impacting the measurements.

Supplementary References

1. McCrum, N. G., Buckley, C. P., Bucknall, C. B. & Bucknall, C. B. *Principles of polymer engineering*. (Oxford University Press, USA, 1997).
